# Significance of Dust Particles, Nanoparticles Radius, Coriolis and Lorentz Forces: The Case of Maxwell Dusty Fluid

**DOI:** 10.3390/nano12091512

**Published:** 2022-04-29

**Authors:** Yanming Wei, Saif Ur Rehman, Nageen Fatima, Bagh Ali, Liaqat Ali, Jae Dong Chung, Nehad Ali Shah

**Affiliations:** 1 School of Science, Xijing University, Xi’an 710123, China; weiyanming@xijing.edu.cn; 2 Department of Mathematics, University of Management and Technology, Lahore 54770, Pakistan; saifurrehman8684@gmail.com (S.U.R.); fatimanageen891@gmail.com (N.F.); 3 Department of Applied Mathematics, Northwestern Polytechnical University, Xi’an 710129, China; baghalisewag@mail.nwpu.edu.cn; 4 Faculty of Computer Science and Information Technology, Superior University, Lahore 54000, Pakistan; 5 Faculty of School of Sciences, Xi’an Technological University, Xi’an 710021, China; liaqat@xatu.edu.cn; 6 Department of Mechanical Engineering, Sejong University, Seoul 05006, Korea; jdchung@sejong.ac.kr

**Keywords:** dusty Maxwell nanofluid, MHD, rotational flow, Darcy–Forchheimer porous medium

## Abstract

This study aimed to analyze the momentum and thermal transport of a rotating dusty Maxwell nanofluid flow on a magnetohydrodynamic Darcy–Forchheimer porous medium with conducting dust particles. Nanouids are the most important source of effective heat source, having many applications in scientific and technological processes. The dust nanoparticles with superior thermal characteristics offer a wide range of uses in chemical and mechanical engineering eras and modern technology. In addition, nanofluid Cu-water is used as the heat-carrying fluid. The governing equations for the two phases model are partial differential equations later transmuted into ordinary ones via similarity transforms. An efficient code for the Runge–Kutta technique with a shooting tool is constructed in MATLAB script to obtain numeric results. The study is compared to previously published work and determined to be perfect. It is observed that the rising strength of the rotating and magnetic parameters cause to recede the *x*- and *y*-axis velocities in the two phase fluid, but the temperature function exhibits an opposite trend. By improving the diameter of nanoparticles $D_{m}$, the axial velocity improves while transverse velocity and temperature show the opposite behaviors. Furthermore, it is reported that the inclusion of dust particles or nanoparticles both cause to decline the primary and secondary velocities of fluid, and also dust particles decrease the temperature.

## 1. Introduction

Non-Newtonian fluids do not follow Newton’s viscosity law, having continuous viscosity regardless of stress. Non-Newtonian nanofluids are employed in various technological, vehicle, and typical housekeeping machinery applications. When external forces influence non-Newtonian fluids, their density might change, resulting in more fluids or solids. To develop innovative non-Newtonian nanofluid features, mathematicians worldwide are exploring novel research models throughout all periods of every day and night. Firstly, Powell and Trying introduced non-Newtonian fluid in 1944. Wang et al. [1] addressed numerical investigation of stream, thermal transport, and the mixing of a highly viscous non-Newtonian fluid was conducted. His work helped develop and optimize industrial heat exchangers for high-viscosity non-Newtonian fluids. Wang et al. [2] deliberated the impact of amphiphilic surfaces on the rheological properties and boundary slip of non-Newtonian fluid. This study might be a useful, practical reference for investigating boundary slip in complex fluids, as well as a critical methodology for examining shear thickening fluids (STFs). Over an extending sheet, Megehed et al. [3] discussed the impacts of heat production on fluid, mass, and thermal transportation of non-Newtonian cross fluid. Rashidi et al. [4] used the multi-step differential transform method (MDTM) to simulate heat transport in the non-Newtonian stream over a porous medium. Sarada et al. [5] investigated the impact of magnetohydrodynamics on the heat transportation of Jefferey and Oldroyd-B liquids. Bilal et al. [6] analyzed the effect of nonlinear viscosity and activation energy for non-Newtonian fluid flow across a slender rotational needle. Maleki et al. [7] discussed the heat transference and fluid flow of a non-Newtonian nanoliquid across a porous surface with suction/injection. In the presence of viscous dissipation, Maleki et al. [8] investigated the heat transportation and fluid flow of non-Newtonian pseudo-plastic nanofluid flow across a porous plate. Jamshed et al. [9] investigated the unsteady flow of Casson nanofluid comprising solar thermal radiation, also including its entropy across a stretching surface. By utilizing the Classical Keller–Box technique, Alazwari et al. [10] examined the entropy production of a stable first grade viscoelastic nanofluid stream across a stretched sheet.

Nanoparticles are tiny particles with a diameter range of (1–100) nm. For the human eye, these are invisible, having chemical and physical properties that differ significantly from their bigger material counterparts. Experts in thermal engineering have embraced the effectiveness of nanofluids due to improvements in heat transmission throughout fluid movement. The nature of the base fluid and nanoparticles underpins the previously described progress. Physical features include nanoparticle concentration and temperature impacts on the mass-to-density ratio and viscosity. Thermal conductivity, specific heat capacity at various concentrations of nanoparticles, and nanoparticles’ size and temperature are some of the thermal characteristics. According to Jama et al. [11], the nanoparticle concentration, pressure drop, friction factor, and nanoparticle radius are some of the described features of nanofluids. A wide range of used nanoparticles are metals, carbides, carbon nanotubes, oxides, etc. By using the Buongiorno model, Revanna et al. [12] presented the impact of MHD fluid flow over a stretching surface with controlled active and passive nanoparticles. Acharya et al. [13] studied the implications of the nanoparticle diameter and heat variations of unsteady radiative nanoliquid flow. Ali et al. [14] investigated the effect of thermal radiation in the MHD nanoliquid stream across the rigid, stagnant plate. Ahmad et al. [15] explored the role of nanoparticles and microorganisms in Homann flow with mass and heat transportation. Almaneea et al. [16] studied the influence of hybrid nanoparticles numerically during chemical reactions with heat and mass transportation. Khan et al. [17] described the efficiency of using nanoparticle suspension in water to minimize heat transfer deterioration (HTD). Ahmad et al. [18] investigated the heat and mass transportation stream of gyrotactic microorganisms and solid nanoparticles across a porous medium numerically. By solving via the finite element method, Nawaz et al. [19] studied thermal increase in the hyperbolic tangent stream with hybrid nanostructures and dust numerically.

Researchers in thermal engineering have embraced the effectiveness of nanofluids due to improvements in heat transmission throughout fluid movement. The nature of the base fluid and nanoparticles underpins the previously described progress. Physical features include nanoparticle concentration and temperature impacts on the mass-to-density ratio and viscosity, thermal conductivity, specific heat capacity at various concentrations of nanoparticles, and the size and temperature of nanoparticles. Nanofluids have multiple features, including nanoparticle concentration, pressure drop, friction factor, and nanoparticle radius.

Because of its unique thermo-physical features and heat transmission efficiency, various researchers and analysts have been driven to examine the magnetohydrodynamic flow of viscous particles across a stretched surface due to its beneficial applications in daily life. Magnetohydrodynamic characteristics of metallic materials, such as plasma, metals, seawater, liquid, and electrolytes, are determined. Magnetic medicines, astrophysical detection, and engineering applications may benefit from MHD. Zhang et al. [20] investigated the effect of Joule thermal and convective conditions on MHD 2D stagnation flow of nanofluid across extending sheet. Koriko et al. [21] explored the bioconvection flow of a magnetohydrodynamic nanofluid in the presence of gyrotactic microorganisms across a sheet. Dadheech et al. [22] analyzed entropy formation on two opposite fluids for radiative sloped magnetohydrodynamics slip flow across a sheet with a thermal source. Abo-Dahab et al. [23] investigated the flow of MHD Casson nanofluid with injection/suction across the non-linearized thermal stretched sheet. Nazeer et al. [24] studied a third-grade magnetohydrodynamic electro-osmotic flow in micron duct theoretically. Salahuddin et al. [25] investigated MHD nanofluid flow behavior and heat formation impact on the preliminary channel. Rehman et al. [26] analyzed heat transportation in MHD Carreau fluid to correspond to various flow regimes theoretically. Zhang et al. [27] studied the heat transportation of nanomaterials through the use of an enclosure employing MHD. Several investigators analyzed MHD flow can be studied in [14,28,29,30].

Due to its wide-ranging uses in sophisticated technologies, engineering, and other branches of science, the flow across a spinning disc has become an advanced subject of research for investigators and engineers. Centrifugal pumps, aeronautical science, engineering areas such as rotating machinery, gas turbine rotors, thermal power-producing systems, air vacuuming devices, and medical equipment are only a few real-world applications. For the very first time, Von Karman studied flow by a rotating disk. Khan et al. [31] discussed numerically the influence of the MHD flow of an Oldroyd-B nanoliquid across a rotating disk with heat and mass transfer. Krishna et al. [32] studied the impact of the magnetic field, thermal radiation, chemical reaction, Hall, and ion slip on the MHD rotational flow of a micro-polar fluid over a moving absorbent sheet. Zubair et al. [33] analyzed the heat transportation of 3D MHD nano liquid rotational flow and shape impacts of Cu nanoparticles across extending sheets. Hayat et al. [34] deliberated the entropy formation and radiation in a rotational stream of viscous nanoliquid between two different geometries. Khan et al. [35] analyzed the rotational flow of Maxwell nanoliquid across a linear/exponential extending surface with double layering. Yacob et al. [36] examined steady 3D rotational flow in nanofluid across extending surfaces in the presence of carbon nanotubes (CNT) nanoparticles. Ali et al. [37] deliberated the Cattaneo–Christov heat flux model using a finite study on the transient MHD rotational flow of Maxwell and tangent hyperbolic nanoliquid across a continuous extending surface numerically. Bagh et al. [38] studied the thermal characteristics of Darcy–Forchheimer fluid flow with a magnetic field effect. Kotresh et al. [39] explored the evaluation of the Arrhenius activation energy in an extended nanoliquid flow across a rotational object, numerically.

Two-phase fluid flow has become more important in the industrial and engineering sectors. One phase is assumed to be dust, while the other is fluid. Large-scale industrial and engineering applications have used dusty nanofluids made of dust substances. As a result, multiple researchers have studied the dusty fluid flow over various surfaces while suspending nanoparticles. Mahanthesh et al. [40] investigated the heat transportation and boundary layer flow in Casson fluid immersed with dust particles across three different shapes (plate, vertical cone, and wedge). Across a vertical plate, Mahanthesh et al. [41] investigated the influence of quadratic convection and thermal radiation on the 2-phase boundary layer flow of nanoliquid, numerically. Mahanthesh et al. [42] studied the dusty Carreau liquid and quadratic transportation of dusty Casson in the presence of thermal radiation with a non-uniform heat source/sink across a stretched surface. In the existence of exponential heat source, the effect of the Hall current on the unsteady heat transference of dusty nanofluid with time-dependent velocity was studied by Mahanthesh et al. [43]. Gireesha et al. [44] examined the impact of the Hall current on the two-phase flow of dusty nanoliquid across the stretching surface by utilizing the KVL model. Mahanthesh et al. [45] discussed the impact of thermal Marangoni convection on two-phase dusty magneto-Casson liquid flow by considering the effect of transpiration cooling. Gireesha et al. [46] explored the effects of the Hall current and heat transportation of dusty liquid with nonlinear thermal radiation across a heated stretched plate.

By analyzing the above literature survey, we deduce that no research has been carried out on the MHD rotating flow of Maxwell fluid with dust particles and nanoparticle radius. Therefore, the prime purpose of the present investigation is to study the outcomes of nanoparticle diameter on the dynamic of Maxwell dusty fluid. Thus, this study explores the consequence of magnetic and Coriolis force effects on dusty fluid flow across extending sheets. The related nonlinear partial differential equations are converted to a system of coupled nonlinear ordinary differential equations by utilizing appropriate similarity modifications. The numerical outcome of the local skin friction coefficient and the local Nusselt number are examined in graphical and tabular form for different physical parameters. The inclusion of dust and nanoparticles with base fluid formed the dusty nanofluid. This numerical report has enormous applications in chemical and mechanical engineering, and others areas, such as the cooling of nuclear reactors, heat exchangers, retrieval of crude oil, waste water treatment, and power technology.

## 2. Mathematical Formulation

**Figure 1:**
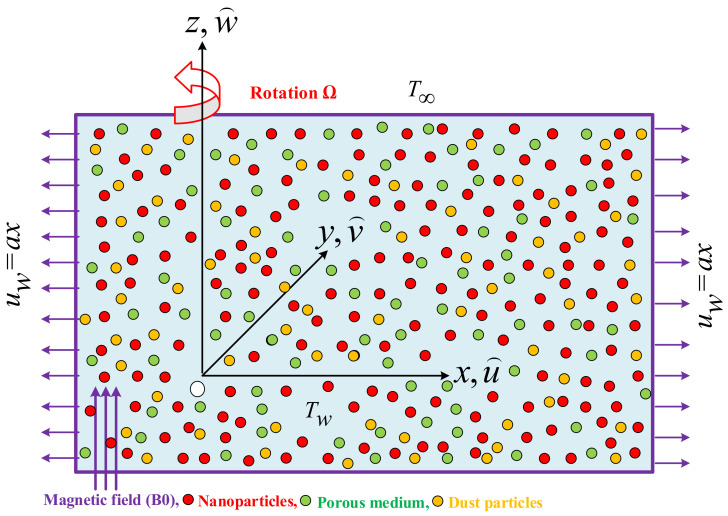
Schematic flow configuration.

A three-dimensional laminar rotating flow of incompressible dusty Maxwell, the dynamics of water conveying copper nanoparticles over a stretching sheet, is assumed. The sheet is stretched in the xy-plane, with fluid placed along the *z*-axis. Fluid rotates about the *z*-axis with constant velocity Ω. Initially, both dust particles and fluid are supposed to be static. The dust particles are considered uniformly sized spheres, and their density is assumed to be constant throughout the stream. Moreover, the ambient temperature of fluid and temperature at the surface are $T_{\infty }$ and $T_{w}$, respectively. Figure 1 demonstrates the coordinate system and flow characteristics. Further, we assume that the thermophysical properties of nanoparticles and base fluid are mentioned in Table 1 and Table 2; there is no slip occurring between the tiny particles, and Cu tiny particles and the host fluid are in thermal equilibrium. The fluid is synthesized as a stable compound and sedimentation of particles is ignored. The mathematical model for the flow of the dusty phase, coupled with the constraints, is as follows, based on these physical assumptions and boundary layer approximations [47,48,49].

(1) $$\begin{array}{c} \frac{\partial \hat{u}}{\partial x}+\frac{\partial \hat{v}}{\partial y}+\frac{\partial \hat{w}}{\partial z}=0,\\ \rho_{nf}(\hat{u}\frac{\partial \hat{u}}{\partial x}+\hat{v}\frac{\partial \hat{u}}{\partial y}+\hat{w}\frac{\partial \hat{u}}{\partial z}-2\Omega \hat{v}+\lambda_{1}[{\hat{u}}^{2}\frac{\partial^{2}\hat{u}}{\partial x^{2}}+{\hat{v}}^{2}\frac{\partial^{2}\hat{u}}{\partial y^{2}}+{\hat{w}}^{2}\frac{\partial^{2}\hat{u}}{\partial z^{2}}+2\hat{u}\hat{v}\frac{\partial^{2}\hat{u}}{\partial x\partial y}+2\hat{v}\hat{w}\frac{\partial^{2}\hat{u}}{\partial y\partial z}\\ +2\hat{u}\hat{w}\frac{\partial^{2}\hat{u}}{\partial x\partial z}-2\Omega \left(\hat{u}\frac{\partial \hat{v}}{\partial x}+\hat{v}\frac{\partial \hat{v}}{\partial y}+\hat{w}\frac{\partial \hat{v}}{\partial z}\right)+2\Omega \left(\hat{v}\frac{\partial \hat{u}}{\partial x}-\hat{u}\frac{\partial \hat{u}}{\partial y})\right]) \end{array}$$

(2) $$\begin{array}{c} =\mu_{nf}\frac{\partial^{2}\hat{u}}{\partial z^{2}}-\sigma B_{0}^{2}\hat{u}-\frac{\nu }{k^{*}}\hat{u}-F\hat{u^{2}}+\frac{KN}{\rho }\left(\hat{u_{p}}-\hat{u}\right),\\ \rho_{nf}(\hat{u}\frac{\partial \hat{v}}{\partial x}+\hat{v}\frac{\partial \hat{v}}{\partial y}+\hat{w}\frac{\partial \hat{v}}{\partial z}+2\Omega \hat{u}+\lambda_{1}[{\hat{u}}^{2}\frac{\partial^{2}\hat{v}}{\partial x^{2}}+{\hat{v}}^{2}\frac{\partial^{2}\hat{v}}{\partial y^{2}}+{\hat{w}}^{2}\frac{\partial^{2}\hat{v}}{\partial z^{2}}+2\hat{u}\hat{v}\frac{\partial^{2}\hat{v}}{\partial x\partial y}+2\hat{v}\hat{w}\frac{\partial^{2}\hat{v}}{\partial y\partial z}\\ +2\hat{u}\hat{w}\frac{\partial^{2}\hat{v}}{\partial x\partial z}+2\Omega \left(\hat{u}\frac{\partial \hat{u}}{\partial x}+\hat{v}\frac{\partial \hat{u}}{\partial y}+\hat{w}\frac{\partial \hat{u}}{\partial z}\right)+2\Omega \left(\hat{v}\frac{\partial \hat{v}}{\partial x}-\hat{u}\frac{\partial \hat{v}}{\partial y}\right)]) \end{array}$$

(3) $$\begin{array}{c} =\mu_{nf}\frac{\partial^{2}\hat{v}}{\partial z^{2}}-\sigma B_{0}^{2}\hat{v}+\frac{KN}{\rho }\left(\hat{v_{p}}-\hat{v}\right), \end{array}$$

(4) $$\begin{array}{c} \left(\rho_{Cp}\right)nf\left(\hat{u}\frac{\partial T}{\partial x}+\hat{v}\frac{\partial T}{\partial y}+\hat{w}\frac{\partial T}{\partial z}\right)=knf\left(\frac{\partial^{2}T}{\partial z^{2}}\right)+\frac{\rho_{p}C_{p}}{\rho_{Cp}\tau_{T}}\left(T_{p}-T\right). \end{array}$$

For dusty particle flow

(5) $$\begin{array}{c} \frac{\partial \hat{u_{p}}}{\partial x}+\frac{\partial \hat{v_{p}}}{\partial y}+\frac{\partial \hat{w_{p}}}{\partial z}=0, \end{array}$$

(6) $$\begin{array}{c} \hat{u_{p}}\frac{\partial \hat{u_{p}}}{\partial x}+\hat{v_{p}}\frac{\partial \hat{u_{p}}}{\partial y}+\hat{w_{p}}\frac{\partial \hat{u_{p}}}{\partial z}=2\Omega \hat{v_{p}}+\frac{KN}{\rho }\left(\hat{u}-\hat{u_{p}}\right), \end{array}$$

(7) $$\begin{array}{c} \hat{u_{p}}\frac{\partial \hat{v_{p}}}{\partial x}+\hat{v_{p}}\frac{\partial \hat{v_{p}}}{\partial y}+\hat{w_{p}}\frac{\partial \hat{v_{p}}}{\partial z}+2\Omega \hat{u}=\frac{KN}{\rho }\left(\hat{v}-\hat{v_{p}}\right), \end{array}$$

(8) $$\begin{array}{c} \hat{u}\frac{\partial T_{p}}{\partial x}+\hat{v}\frac{\partial T_{p}}{\partial y}+\hat{w}\frac{\partial T_{p}}{\partial z}=\frac{c_{p}}{c_{m}\tau_{T}}\left(T-T_{p}\right). \end{array}$$

The dimensional boundary conditions are

(9) $$\begin{array}{c} \left.\begin{array}{c} \hat{u}=u_{w}=ax,\hat{v}=0,\hat{w}=0,\;T=T_{w},\;at\;z=0,\;\\ \hat{u_{p}}=u_{w}=ax,\hat{v_{p}}=0,\hat{w}=0\;T_{p}=T_{w},\;at\;z=0,\\ \hat{u}\to 0,\hat{v}\to 0,\hat{w}\to w\;\hat{T}\to T_{\infty },\;\;as\;z\to \infty ,\;\\ \hat{u_{p}}\to 0,\hat{v_{p}}\to 0,\hat{w_{p}}\to w\;\hat{T_{p}}\to T_{\infty },\;as\;z\to \infty .\; \end{array}\right\} \end{array}$$

By introducing similarity transform [50]

(10) $$\begin{array}{c} \left.\begin{array}{c} \hat{u}=axf^{\prime }\left(\eta \right),\;\hat{v}=axg\left(\eta \right),\;\hat{w}=-\sqrt{a\nu }f\left(\eta \right),\;\eta ={\left(\frac{a}{\nu }\right)}^{\frac{1}{2}}z,\\ \theta \left(\eta \right)=\frac{T-T_{\infty }}{T_{w}-T_{\infty }},\;\\ \hat{u_{p}}=axF^{\prime }\left(\eta \right),\;\hat{v_{p}}=axG\left(\eta \right),\;\hat{w_{p}}=-\sqrt{a\nu }F\left(\eta \right),\;\eta =\sqrt{\frac{a}{\nu }}z\\ \theta_{p}\left(\eta \right)=\frac{T_{p}-T_{\infty }}{T_{w}-T_{\infty }},\;\; \end{array}\right\} \end{array}$$

where η stands for similarity variable.

Equation (1) is identically fulfilled when the above-mentioned transformations are substituted, whereas Equations (2)–(8) have the following structure:

(11) $$\begin{array}{c} \frac{A_{1}}{A_{2}}F^{\prime \prime \prime }+FF^{\prime \prime }+2\lambda \left(G-\beta_{m}FG^{\prime }\right)+\lambda \left(2FF^{\prime }F^{\prime \prime }-F^{2}F^{\prime \prime \prime }\right)-\frac{A_{3}}{A_{2}}MF^{\prime }-K_{p}F^{\prime }-\left(1+Fr\right)F^{\prime 2}+\frac{\Gamma_{v}\gamma_{v}}{A_{2}}\left(F_{p}^{\prime }-F^{\prime }\right)=0, \end{array}$$

(12) $$\begin{array}{c} \frac{A_{1}}{A_{2}}G^{\prime \prime }+F^{\prime }G-FG^{\prime }-K_{p}G+2\lambda \left(F^{\prime }+\beta_{m}\left(F^{\prime 2}-FF^{\prime \prime }+G^{2}\right)\right)+\beta_{m}\left(2FF^{\prime }G^{\prime }-F^{2}G^{\prime \prime }\right)-\frac{A_{3}}{A_{2}}MG+\frac{\Gamma_{v}\gamma_{v}}{A_{2}}\left(G_{p}-G\right)=0, \end{array}$$

(13) $$\begin{array}{c} \frac{A_{4}}{A_{5}}\theta^{\prime \prime }+Prf\theta^{\prime }+\frac{Pr}{A_{5}}\gamma_{t}.\beta_{t}\left(\theta_{p}-\theta \right)=0, \end{array}$$

For dusty phase

(14) $$\begin{array}{c} F_{p}^{\prime 2}-F_{p}F_{p}^{\prime \prime }-2\lambda G_{p}+\gamma_{v}.\Gamma_{v}\left(F_{p}^{\prime }-F^{\prime }\right)=0, \end{array}$$

(15) $$\begin{array}{c} F_{p}^{\prime }G_{p}-F_{p}G_{p}^{\prime }+2\lambda F_{p}^{\prime }+\gamma_{v}.\Gamma_{v}\left(G_{p}-G\right)=0, \end{array}$$

(16) $$\begin{array}{c} F_{p}\theta_{p}^{{}^{\prime }}+\gamma_{t}.\beta_{t}\left(\theta -\theta_{p}\right)=0, \end{array}$$

with boundary constraints,

(17) $$\begin{array}{c} \left.\begin{array}{c} F\left(\eta \right)=0,\;G\left(\eta \right)=0,\;F^{\prime }\left(\eta \right)=1,\theta \left(\eta \right)=1,\;at\;\eta =0,\;\\ F_{p}\left(\eta \right)=F\left(\eta \right),\;G_{p}\left(\eta \right)=G\left(\eta \right),\;F_{p}^{\prime }\left(\eta \right)=1,\theta_{p}\left(\eta \right)=1,\;at\;\eta =0,\\ F^{\prime }\left(\eta \right)\to 0,G^{\prime }\left(\eta \right)\to 0,\;\theta \left(\eta \right)\to 0,\;at\;\eta \to \infty .\;\\ F_{p}^{\prime }\left(\eta \right)\to 0,G_{p}^{\prime }\left(\eta \right)\to 0,\;\theta_{p}\left(\eta \right)\to 0,\;at\;\eta \to \infty .\; \end{array}\right\} \end{array}$$

where $A_{1}=1+2.5\phi +4.5\left[\frac{1}{\frac{h}{d_{p}}\left(2+\frac{h}{d_{p}}\right){\left(1+\frac{h}{d_{p}}\right)}^{2}}\right]$, $A_{2}=1-\phi +\phi \frac{\rho_{s}}{\rho_{f}}$, $A_{3}=\left[1+\frac{3(\frac{\sigma_{s}}{\sigma_{f}}-1)\phi }{\left(\frac{\sigma_{s}}{\sigma_{f}}\right)-\left(\frac{\sigma_{s}}{\sigma_{f}}-1\right)\phi }\right]$, $A_{4}=\frac{k_{s}+2k_{f}-2\phi \left(k_{f}-k_{s}\right)}{k_{s}+2k_{f}+\phi \left(k_{f}-k_{s}\right)}$, and $A_{5}=1-\phi +\phi \frac{{\left(\rho C_{p}\right)}_{s}}{{\left(\rho C_{p}\right)}_{f}}$ declared for the $Pr=\frac{\mu_{f}{\left(cp\right)}_{f}}{k_{f}}$ represent the Prandtl number, $\lambda =\frac{\Omega }{a}$ stands for rotational flow, $\gamma_{v}=\frac{1}{\tau_{v}c}$ elaborates the fluid particle interaction parameter for velocity, $\gamma_{v}=\frac{Nm}{\rho }$ declares for the mass concentration of dusty granules, $\Gamma_{t}=\frac{c_{p}}{c_{m}}$ represents the ratio of specific heat, $\beta_{t}=\frac{1}{a\tau_{T}}$ constitutes the temperature interaction, $M=\sqrt{\frac{\sigma \;B_{o}^{2}}{\rho_{nf}a}}$ signifies the magnetic field parameter, $\beta_{m}$ is the Maxwell parameter, $Fr=\frac{C_{b}}{\sqrt{k^{*}}}$ represents the inertia coefficient, and $K_{p}=\frac{\nu_{f}}{k^{*}a}$ represents the porous medium.

## 3. Physical Quantities

The important physical quantities of interest are discussed in this section. The Nusselt coefficients $Nu_{x}$ are given as

(18) $$\begin{array}{c} Nu_{x}=\frac{xq_{w}}{k_{f}\left(T_{w}-T_{\infty }\right)}, \end{array}$$

where $q_{w}$ is the heat flux,

(19) $$\begin{array}{c} q_{w}=-k_{nf}{\left(\frac{\partial T}{\partial z}\right)}_{z=0}, \end{array}$$

we derive the following formulas,

(20) $$\begin{array}{c} Re_{x}^{\frac{-1}{2}}Nu_{x}=\frac{k_{nf}}{k_{f}}\left(-\theta^{\prime }\left(0\right)\right), \end{array}$$

The local Reynolds number $Re_{x}=\frac{u_{w}^{2}}{c\nu }$.

## 4. Solution Procedure

**Figure 2:**
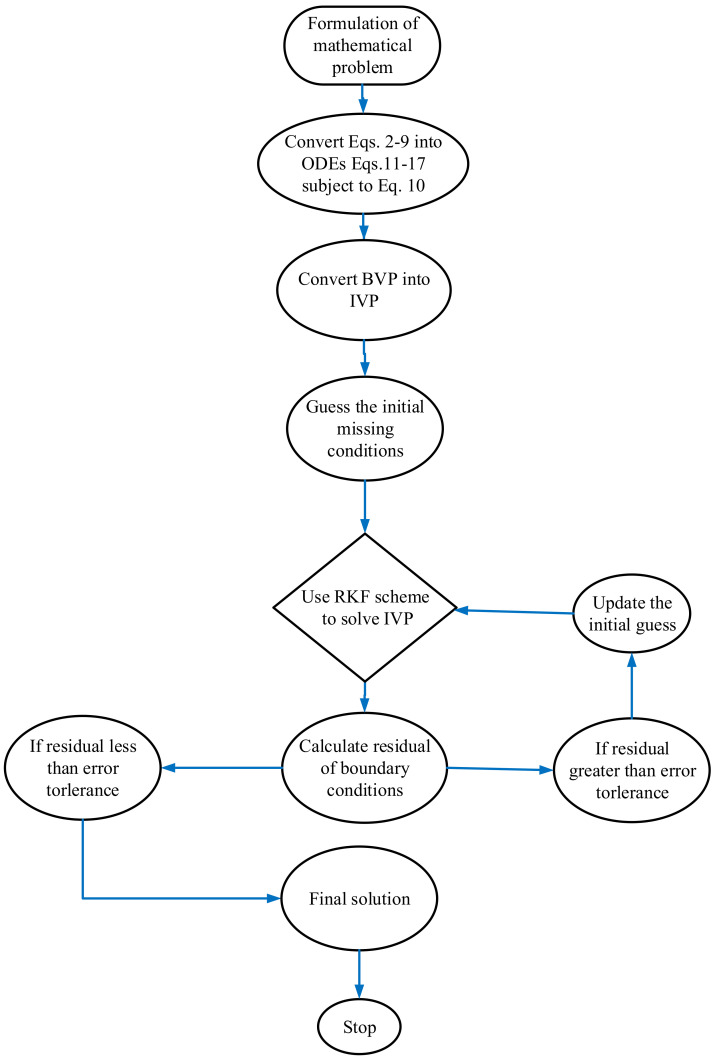
Flow chart.

A system of ordinary differential equations represents the flow model. Algorithms (11)–(16) are numerically solved using the effectiveness and strength of numerical computing in the Runge-Kutta approach using MATLAB. Refs. [52,53] provides more details on this solution approach (see Figure 2). The graphical and numerical results show the velocity and temperature fields as different physical factors. The Runge–Kutta technique is used to transform the system of ODEs (11)–(16) into first-order ODEs for a solution:

$s_{1}^{\prime }=s_{2},$

$s_{2}^{\prime }=s_{3},$

$s_{3}^{\prime }=\frac{\left(-1\right)}{\left(\frac{A_{1}}{A_{2}}-\lambda s_{1}^{2}\right)}\left[s_{1}s_{3}+2\lambda \left(s_{4}-\beta_{m}s_{1}s_{5}\right)+2\lambda s_{1}s_{2}s_{3}-\frac{A_{3}}{A_{2}}Ms_{2}-K_{p}s_{2}-\left(1+Fr\right)s_{2}^{2}+\frac{\Gamma_{v}\gamma_{v}}{A_{2}}\left(s_{9}-s_{2}\right)\right],$

$s_{4}^{\prime }=s_{5},$

$s_{5}^{\prime }=\frac{\left(-1\right)}{\left(\frac{A_{1}}{A_{2}}-\beta_{m}s_{1}^{2}\right)}\left[s_{1}s_{4}-s_{1}s_{5}-K_{p}s_{4}+2\lambda \left(s_{2}+\beta_{m}\left(s_{2}^{2}-s_{1}s_{3}+s_{4}^{2}\right)\right)+\beta_{m}\left(s_{1}s_{2}s_{5}\right)-\frac{A_{3}}{A_{2}}Ms_{4}+\frac{\Gamma_{v}\gamma_{v}}{A_{2}}\left(s_{10}-s_{4}\right)\right],$

$s_{6}^{\prime }=s_{7},$

$s_{7}^{\prime }=\frac{\left(-A_{5}\right)}{A_{4}}\left[Prs_{1}s_{7}+\frac{Pr}{A_{5}}\gamma_{t}\beta_{t}\left(s_{11}-s_{6}\right)\right],$

$s_{8}^{\prime }=s_{9}$

$s_{9}^{\prime }=\frac{\left(-1\right)}{s_{8}}\left[s_{9}^{2}-2\lambda s_{10}+\Gamma_{v}\gamma_{v}\left(s_{9}-s_{2}\right)\right],$

$s_{10}^{{}^{\prime }}=\frac{\left(-1\right)}{s_{8}}\left[s_{9}s_{10}+2\lambda s_{9}+\Gamma_{v}\gamma_{v}\left(s_{10}-s_{4}\right)\right],$

$s_{11}^{{}^{\prime }}=\frac{\left(-1\right)}{s_{8}}\left[\beta_{t}\gamma_{t}\left(s_{6}-s_{11}\right)\right],$

The corresponding boundary conditions are as follows:

$$\begin{array}{l} s_{1}=0,\;s_{2}=1,\;s_{4}=0,\;s_{6}=1,\;at\;\eta =0,\;\\ s_{8}=0,\;s_{9}=1,\;s_{1}1=0,\;s_{13}=1,\;at\;\eta =0,\;\\ s_{2}\to 0,\;s_{5}\to 0,\;s_{6}\to 0,\;as\;\eta \to \infty .\;\\ s_{9}\to 0,\;s_{12}\to 0,\;s_{13}\to 0,\;as\;\eta \to \infty .\; \end{array}$$

## 5. Results and Discussion

This research aims to investigate Maxwell nanofluid flow and melting thermal transport across a stretched surface with fluid–particle suspension. The Runge–Kutta approach solves the governed highly nonlinear expressions with associated conditions coded in MATLAB. The numerical code validation is obtained by comparing the numerical outcomes of the current and past results of skin friction [47,48] and Nusselt numbers. Our findings are obtained to be in good agreement with them (see Table 3 and Table 4). The impact of parameters on momentum, thermal, and concentration layers in dual fluid and dusty phases. We take values of parameters as $\beta_{m}=0.2,M=0.5,\lambda =0.3,Pr=2.0,K_{p}=0.3,Fr=1.0,\Gamma_{v}=0.2,\gamma_{t}=0.2,\beta_{v}=0.2,\beta_{t}=0.1$.

**Figure 3:**
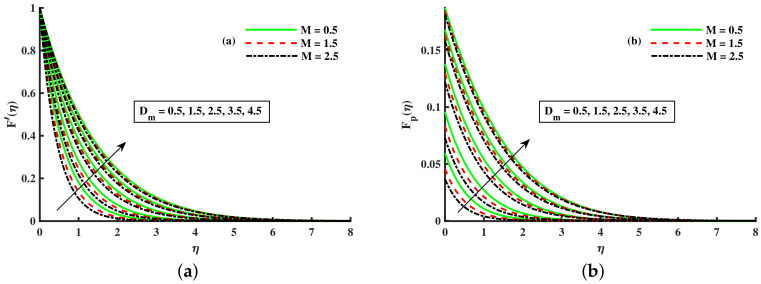
Fluctuation of *M* and $D_{m}$ on $F^{\prime }\left(\eta \right)$ (**a**) and the effect of *M* and $D_{m}$ on $F_{p}\left(\eta \right)$ (**b**).

**Figure 4:**
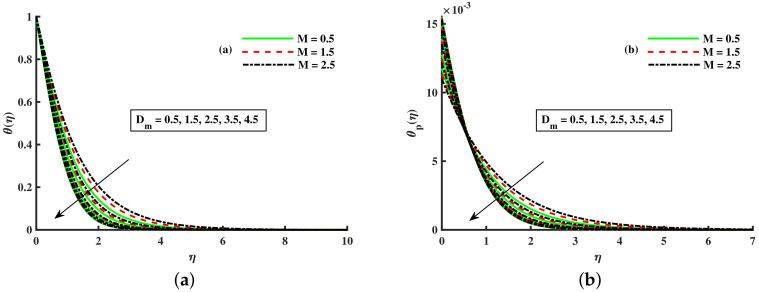
Fluctuation of *M* and $D_{m}$ on θ(η) (**a**) and the effect of *M* and $D_{m}$ on $\theta_{p}\left(\eta \right)$ (**b**).

**Figure 5:**
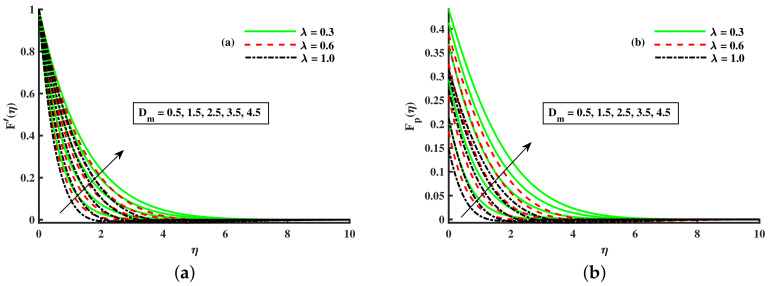
Fluctuation of λ and $D_{m}$ on $F^{\prime }\left(\eta \right)$ (**a**) and the effect of λ and $D_{m}$ on $F_{p}\left(\eta \right)$ (**b**).

**Figure 6:**
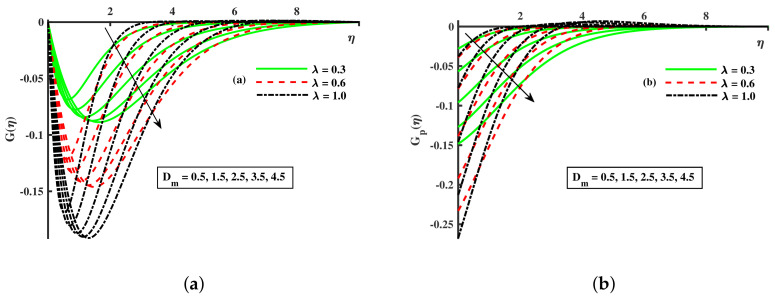
Fluctuation of λ and $D_{m}$ on G(η) (**a**) and the effect of λ and $D_{m}$ on $G_{p}\left(\eta \right)$ (**b**).

**Figure 7:**
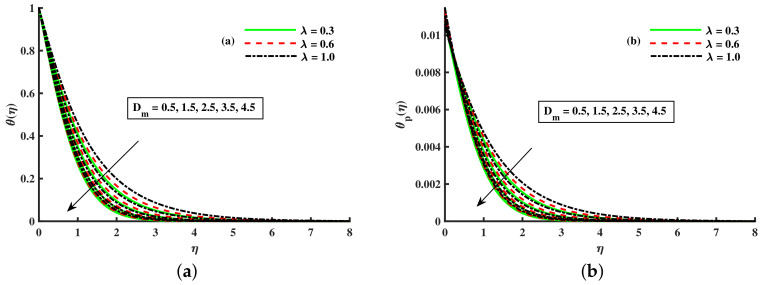
Fluctuation of λ and $D_{m}$ on θ(η) (**a**) and the effect of λ and $D_{m}$ on $\theta_{p}\left(\eta \right)$ (**b**).

Figure 3a,b and Figure 4a,b depict that the momentum boundary layer of the transport phenomenon rises as the nanoparticles’ radius increases. Meanwhile, an increase in the nanoparticles radius causes an increase in friction throughout the layer along the rotating surface; this is because, as Namburu et al. [54] pointed out, increasing the diameter of nanoparticles, the velocity increases during the thermal boundary layer show opposite behavior for fluid and dust particles. On the other hand, the velocity and thermal boundary layer are for various inputs of the magnetic parameter. Here, F′(η) signifies momentum in the *x*-direction for the fluid phase, and $F_{p}\left(\eta \right)$ demonstrates the momentum boundary layer in the *x*-direction for the dust phase, respectively. It can be seen that the velocity lowered while the temperature boundary layer rose with improving the *M* inputs. The influence of a magnetic field on an electrically conducting fluid causes the resistive force that tends to reduce the momentum while increasing the thermal boundary layer. Because of this, the magnetic field impact has a wide range of control-based applications, including magnetohydrodynamic ion propulsion, electromagnetic metal casting, magnetohydrodynamic power production, etc. The velocity and thermal boundary layer for different inputs of the diameter of nanoparticles are portrayed in Figure 5 and Figure 6. Here, F′(η) signifies the momentum along the *x*-axis while G(η) is the momentum along the *y*-axis for the fluid phase, and $F_{p}\left(\eta \right)$ deliberates the momentum of the *x*-axis while $G_{p}\left(\eta \right)$ represents the momentum in the *y*-axis for the dust particles, respectively. It is observed that the momentum profile decreased. The reduced viscosity of water-based nanofluid caused due to a larger copper nanoparticles radius leads to a rise in momentum at all levels of energy flow due to the concentration gradient. When the energy flow owing to the concentration gradient is sufficient in magnitude, a drop occurs in temperature distribution over the domain. The other side shows the impact of the rotation parameter λ on the momentum and thermal boundary layer of the fluid and dust particles. The velocity along the *x*-direction declines and in the *y*-direction, it enhances fluid and dust particles with improving λ. Thus, an improving λ thermal distribution of fluid and dust particles is depicted in Figure 7a,b.

**Figure 8:**
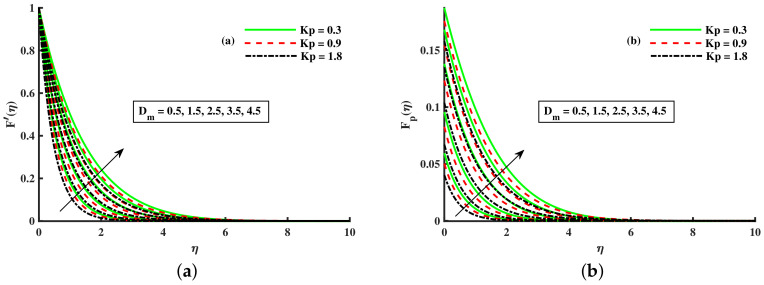
Fluctuation of Kp and $D_{m}$ on $F^{\prime }\left(\eta \right)$ (**a**) and the effect of Kp and $D_{m}$ on $F_{p}\left(\eta \right)$ (**b**).

**Figure 9:**
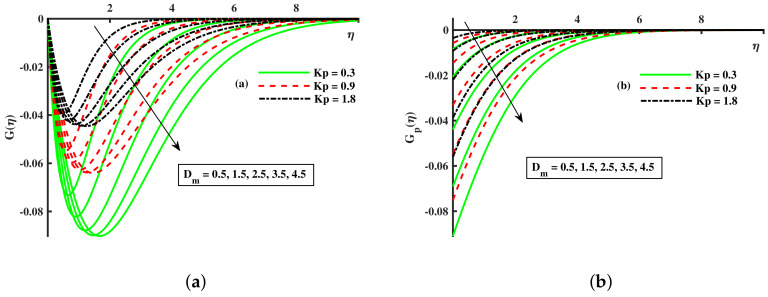
Fluctuation of Kp and $D_{m}$ on G(η) (**a**) and the effect of Kp and $D_{m}$ on $G_{p}\left(\eta \right)$ (**b**).

**Figure 10:**
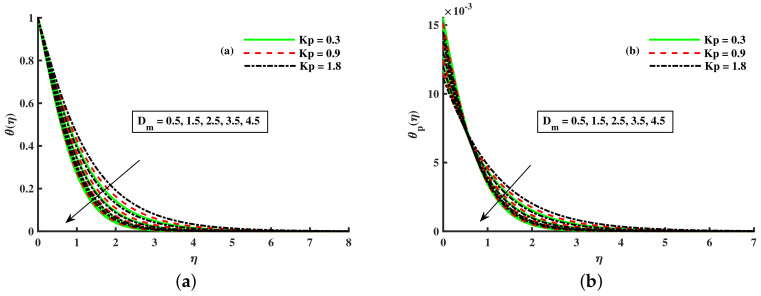
Fluctuation of Kp and $D_{m}$ on θ(η) (**a**) and the effect of Kp and $D_{m}$ on $\theta_{p}\left(\eta \right)$ (**b**).

**Figure 11:**
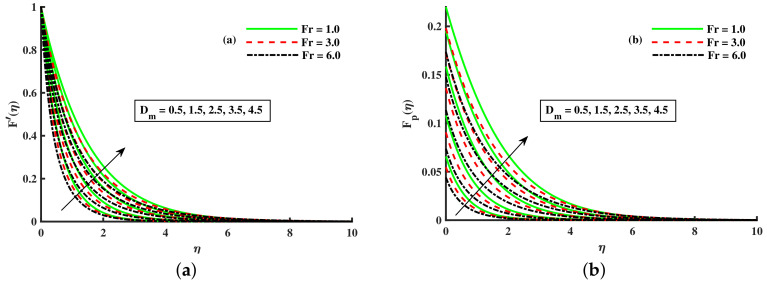
Fluctuation of Fr and $D_{m}$ on $F^{\prime }\left(\eta \right)$ (**a**) and the effect of Fr and $D_{m}$ on $F_{p}\left(\eta \right)$ (**b**).

**Figure 12:**
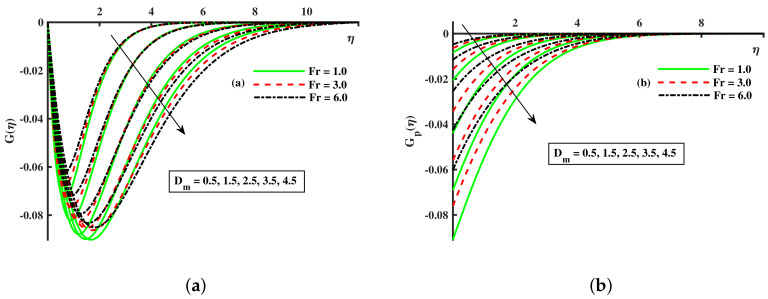
Fluctuation of Fr and $D_{m}$ on G(η) (**a**) and the effect of Fr and $D_{m}$ on $G_{p}\left(\eta \right)$ (**b**).

**Figure 13:**
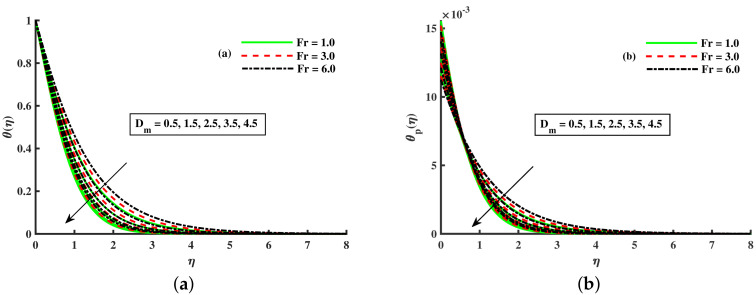
Fluctuation of Fr and $D_{m}$ on θ(η) (**a**) and the effect of Fr and $D_{m}$ on $\theta_{p}\left(\eta \right)$ (**b**).

Figure 8, Figure 9 and Figure 10 are depicted for the influence of the nanoparticles’ diameter. It is noticed that improving the $D_{m}$ enhances the axial velocity of fluid and dust particles and depreciates for transverse velocity and temperature. On the other hand, it can be seen that by improving the inputs of the $K_{p}$ depreciation in axial momentum boundary layer thickness, and the rise in the transverse momentum boundary layer, caused due to $K_{p}$$\left(K_{p}=\frac{\nu }{K^{*}a}\right)$ is converse to the permeability $k^{*}$ of the porous medium. The higher values of $K_{p}$ mean lower $k^{*}$ and hence higher resistance is offered to the flow to the axial velocities ($F\prime \left(\eta \right),F_{p}\left(\eta \right)$) and enhances the transverse velocities ($G\left(\eta \right),G_{p}\left(\eta \right)$), and thermal boundary layers ($\theta \left(\eta \right),\theta_{p}\left(\eta \right)$). Similarly, the local inertia parameter Fr on velocity for fluid and dust diminished for growing values of Fr, caused due to inertia coefficient, is a direct relation to the medium porosity and drag force. So when Cb enhances, the liquid resistive force is stronger, and the axial velocity is lower for the fluid and dust phases. It rises in transverse velocity and temperature for fluid and dust particles. The influence of fluid axial velocity ($F^{\prime }\left(\eta \right),F_{p}\left(\eta \right)$), transverse velocity ($G\left(\eta \right),G_{p}\left(\eta \right)$), and temperature distribution of Maxwell nanofluid and dust phase, θ and $\theta_{p}$, are depicted in Figure 11, Figure 12 and Figure 13.

**Figure 14:**
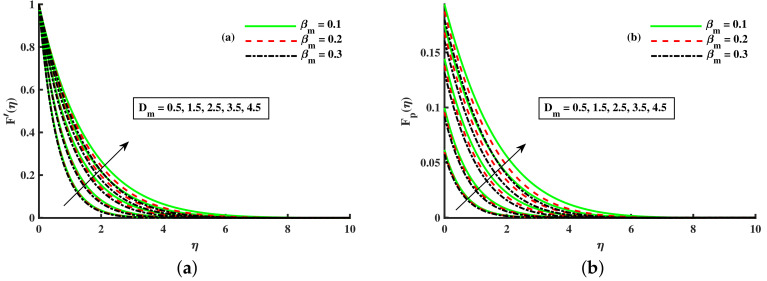
Fluctuation of $\beta_{m}$ and $D_{m}$ on $F^{\prime }\left(\eta \right)$ (**a**) and the effect of $\beta_{m}$ and $D_{m}$ on $F_{p}\left(\eta \right)$ (**b**).

**Figure 15:**
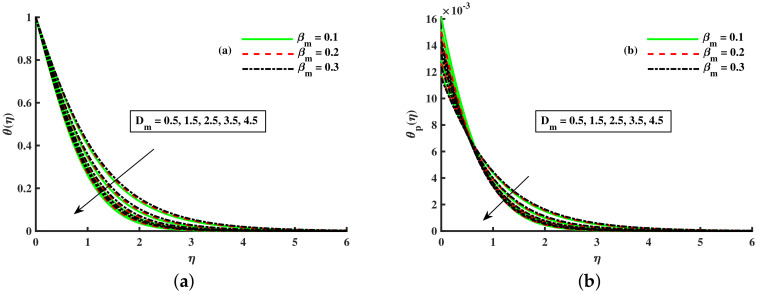
Fluctuation of $\beta_{m}$ and $D_{m}$ on θ(η) (**a**) and the effect of $\beta_{m}$ and $D_{m}$ on $\theta_{p}\left(\eta \right)$ (**b**).

**Figure 16:**
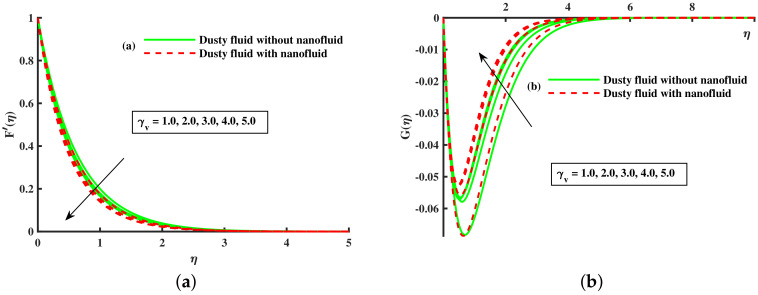
Fluctuation of $\gamma_{v}$ on $F^{\prime }\left(\eta \right)$ (**a**) and the effect of $\gamma_{v}$ on G(η) (**b**).

**Figure 17:**
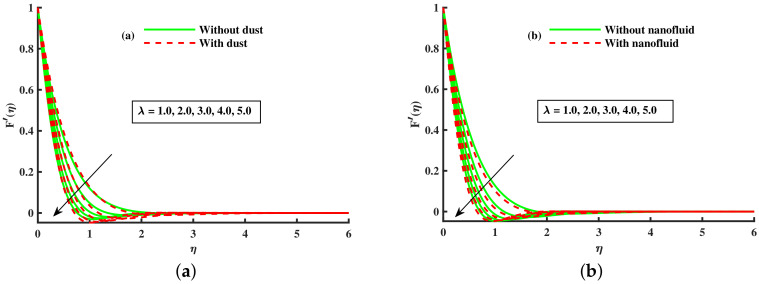
Fluctuation of λ on $F^{\prime }\left(\eta \right)$ (**a**) and effect of λ on $F^{\prime }\left(\eta \right)$ (**b**).

**Figure 18:**
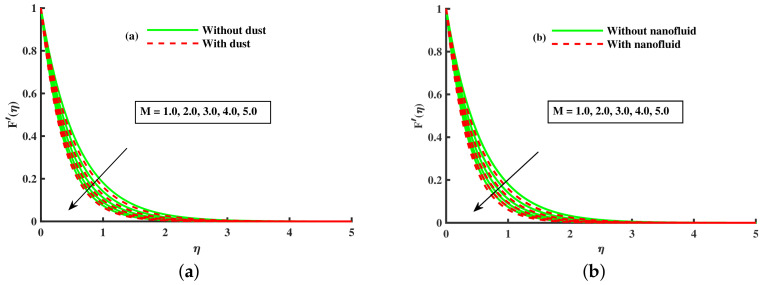
Fluctuation of *M* on $F^{\prime }\left(\eta \right)$ (**a**) and effect of *M* on $F^{\prime }\left(\eta \right)$ (**b**).

Figure 14a,b portrays the momentum boundary layer and thermal boundary layer for various inputs of $\beta_{m}$. The axial velocity depreciation occurs with enhanced $\beta_{m}$. It is caused because the higher values of $\beta_{m}$ present a stronger viscous force, which causes a depreciation in the flow and, hence, velocity ($F^{\prime }\left(\eta \right),F_{p}\left(\eta \right)$) decline. We observed that distributions are tilted toward the boundary when β is enhanced. Figure 15a,b depicts the curve of thermal boundary layers $\left(\theta \left(\eta \right),\theta_{p}\left(\eta \right)\right)$ for different inputs of $\beta_{m}$. It is observed that the penetration depth of the thermal boundary layers is enhanced by improving $\beta_{m}$. Figure 16a,b depicts that the magnitude of the momentum boundary layer of the transport phenomenon decreases as the fluid particle interaction parameter ($\gamma_{v}$) increases. Further, it is noted from this figure that the magnitude of the primary and secondary dusty fluid velocities are lesser in the dusty nanofluid case as compared to the dusty phase when nanoparticles are ignored. Figure 17a,b and Figure 18a,b show the influence of *M* and λ on the dynamic of the base fluid velocity with and without dust, and nanofluid, respectively. It is observed that the momentum profile is decreased against higher input of magnetic and rotating parameters, but the momentum profile exhibits more decline when dust particles and nanoparticles are incorporated in the base fluid.

**Figure 19:**
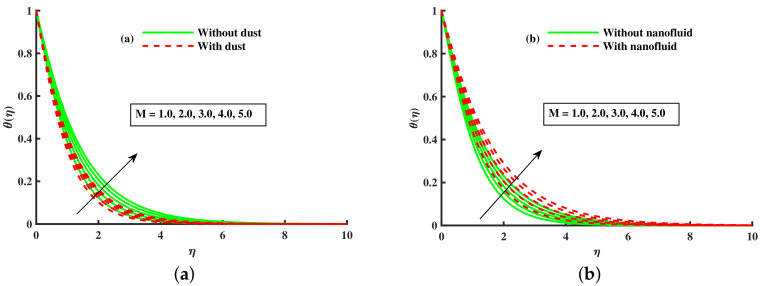
Fluctuation of *M* on θ(η) (**a**) and effect of *M* on θ(η) (**b**).

**Figure 20:**
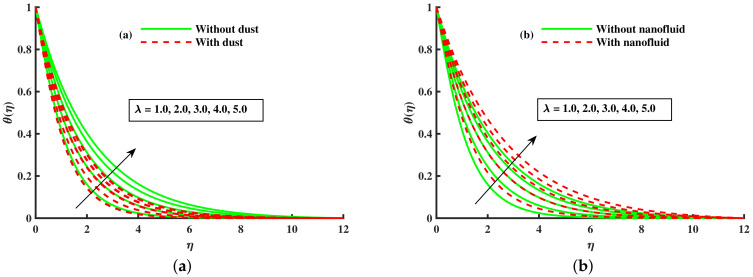

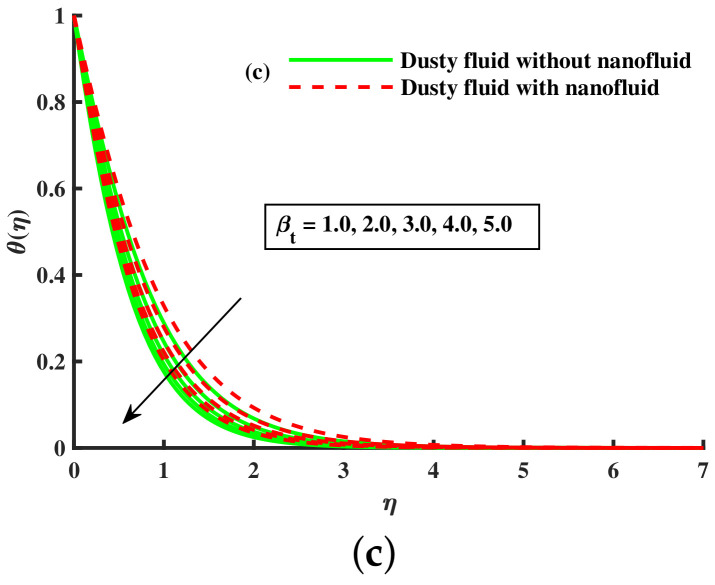
Fluctuation of *M* with nanofluid on θ(η) (**a**) effect of *M* on θ(η) (**b**), and $\beta_{t}$ on θ(η) (**c**).

**Figure 21:**
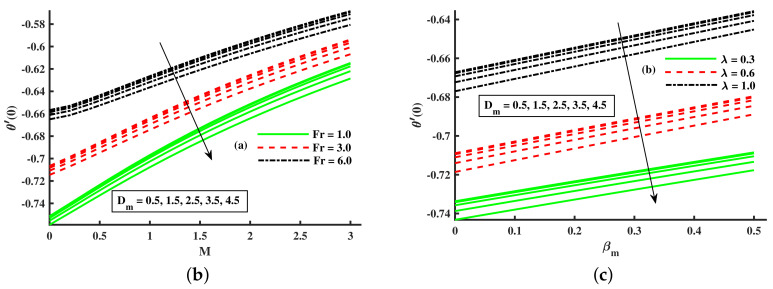
Effect of Fr, $D_{m}$, *M* on Nusselt number (**a**) effect of λ, $D_{m}$, $\beta_{m}$ on Nusselt number (**b**).

From Figure 19 and Figure 20a–c, the influence of *M*, λ, & $\beta_{t}$ on the characteristic of base fluid temperature (θ(η)) is discussed. We discuss two separate cases (with and without dust, and with and without nanofluid) in these two diagrams. We can see from these two diagrams (Figure 19 and Figure 20a,b) that the base fluid temperature rises against the growing strength of magnetic and rotating parameters values, and the nanofluid phase also raises the base fluid temperature, but the opposite trend is observed for the dust particles phase. Physically, the addition of nanoparticles enhances the temperature due to the extraordinary property to enhanced the base fluid temperature. Experts in thermal engineering have embraced the effectiveness of nanofluids due to the improvements in heat transmission throughout fluid movement. Furthermore, Figure 20c shows the decline in the thermal boundary against higher values of $\beta_{t}$ and nanofluid phase. The nanofluid phase enhances the thermal boundary layer as compared to that without the nanoparticles phase. Physically, the addition of nanoparticles enhances the temperature due to the extraordinary property to enhanced the base fluid temperature. Figure 21a,b portrays the Nusselt number for various inputs of $\beta_{m}$, magnetic (M), and rotating (λ). The Nusselt number is decreased negatively against higher values for higher values of $\beta_{m}$, magnetic (M), and rotating (λ), but the opposite behavior is reported against the nanoparticles radius.

## 6. Conclusions

An effort has been made to investigate the importance of raising the radius of nanoparticles and heat flux due to the thermal boundary gradient in the dynamic of the Maxwell nanofluid across the stretched sheet. The non-dimensional boundary value problem is resolved numerically. The code of the Runge–Kutta method is developed in a MATLAB script. Based on the results, it is reasonable to conclude the following:

- Throughout all levels of energy flux caused to the concentration gradient, a decrease in the viscosity of water-based nanofluid due to a larger radius of copper nanoparticles produces an increase in the momentum boundary layer.
- It is observed that the growing strength of the rotating and magnetic parameters cause the *x* and *y* axis velocities in the two phase fluid to recede, but the temperature function exhibits an opposite trend.
- The higher input in the porosity and Forchheimer number causes a decline in the magnitude of the *x*- and *y*-axis velocities in the two-phase fluid, but the temperature is upgraded.
- A significant decline in the thermal boundary layer over the domain, caused due to enhancing the radius of Copper nanoparticles, is feasible when the heat flux caused to the concentration gradient is sufficiently higher in magnitude.
- The inclusion of dust particles or nanoparticles both cause to decline the primary and secondary velocities of fluid, and also dust particles decrease the temperature, but nanoparticles enhance the base fluid temperature.
- The Nusselt number is decreased negatively against higher values for higher values of $\beta_{m}$, magnetic (M), and rotating (λ), but the opposite behavior is reported against the nanoparticles radius.
- The present results are compared with the past literature to validate the results.

## Figures and Tables

**Table 1:** Thermophysical properties of nanofluids are represented by mathematical relationships [51].

Properties	Nanofluid
Density	$\rho_{nf}=1-\phi \rho_{f}+\phi \rho_{s}$,
Nanofuid’s Dynamic Viscosity to Base Fluid’s Dynamic Viscosity Ratio	$\frac{\mu_{nf}}{\mu_{bf}}=1+2.5\phi +4.5\left[\frac{1}{\frac{h}{D_{m}}\left(2+\frac{h}{D_{m}}\right){\left(1+\frac{h}{D_{m}}\right)}^{2}}\right]$ ,
Thermal Conductivity	$\frac{k_{nf}}{k_{f}}=\frac{k_{s}+2k_{f}-2\phi \left(k_{f}-k_{s}\right)}{k_{s}+2k_{f}+\phi \left(k_{f}-k_{s}\right)}$ ,
Electrical Conductivity	$\frac{\sigma_{nf}}{\sigma_{f}}=\left[1+\frac{3(\frac{\sigma_{s}}{\sigma_{f}}-1)\phi }{\left(\frac{\sigma_{s}}{\sigma_{f}}\right)-\left(\frac{\sigma_{s}}{\sigma_{f}}-1\right)\phi }\right]$,
Heat Capacity	${\left(\rho_{cp}\right)}_{nf}=1-\phi {\left(\rho_{Cp}\right)}_{f}+\phi {\left(\rho_{Cp}\right)}_{s},$

**Table 2:** Thermophysical properties of nanoparticles Cu and base fluid water.

Physical Properties	Cu	Water/Base Fluid
density (ρ)	8933	997.1
specific heat ($C_{p}$)	385	4179
Thermal conductivity (k)	401	0.613
Electrical Conductivity (σ)	5.96 × $10^{-7}$	5.5 × $10^{-6}$

**Table 3:** Comparison for skin friction coefficient for various inputs of λ,β,Fr when ignored other parameters.

λ	β	Fr	Rashid et al. [47]	Tayyab et al. [48]	Present Results
0.1	0.5	1.0	1.41806	1.4180632	1.41806
0.5			1.53031	1.530305	1.53031
1.0			1.67153	1.671530	1.67153
0.2	1.0		1.58724	1.587236	1.58703
	2.0		1.86267	1.862673	1.86268
	3.0		2.10932	2.109315	2.10932
0.1	0.5	1.0	1.36352	1.363524	1.36352
		2.0	1.58502	1.585020	1.58502
		4.0	1.96195	1.961949	1.96190

**Table 4:** Comparison for Nusselt number for different inputs of λ,β,Pr,Fr when ignoring other parameters.

λ	β	Pr	Fr	Rashid et al. [47]	Tayyab et al. [48]	Present Results
0.0	0.5	1.0	1.0	0.508972	0.508972	0.50897
1.0				0.485852	0.4858519	0.48585
2.0				0.457520	0.4575203	0.45752
0.2	0.1			0.542198	0.541983	0.54198
3	0.5			0.506965	0.5069650	0.50696
2.0	0.9			0.467794	0.4677941	0.46779
1.0	0.5	2.0		0.811336	0.8113353	0.81133
		3.0		1.064611	1.0646115	1.06461
		4.0		1.278977	1.2789778	1.27897
		1.0	0.0	0.844615	1.8446157	0.84461
			2.0	0.783173	0.7831728	0.78317
			3.0	0.734272	0.7580794	0.75807

## Data Availability

No data were used to support this study.

## Nomenclature

$T_{\infty }$	Ambient temperature, (K)
*F*	Co-efficient of inertia of porous material
*N*	Dust particle number constant
$\hat{u}$	Fluid velocity components along the *x*-axis (m${}^{2}$ s${}^{-1}$)
$\hat{v}$	Fluid velocity components along the *y*-axis (m${}^{2}$ s${}^{-1}$)
$\hat{w}$	Fluid velocity components along the *y*-axis (m${}^{2}$ s${}^{-1}$)
$B_{0}$	Magnetic field strength
*m*	Mass of dust particle (kg)
$Nu_{x}$	Nusselt number
$k^{*}$	Permeability of porous medium
*a*	positive constant
$c_{p}$	Specific heat capacity of the fluid (m s${}^{-2}$)
Cp	Specific heat and constant pressure, (J kg${}^{-1}$ K${}^{-1})$
$c_{m}$	Specific heat of dust particle
*K*	Stoke’s drag constant
*T*	Temperature of fluid (K)
$T_{w}$	Temperature at the surface, (K)
$T_{p}$	Temperature of the dust particle (K)
$k_{f}$	Thermal conductivity, (W m${}^{-1}$ K${}^{-1}$)
$\hat{u_{p}},\hat{v_{p}},\hat{w_{p}}$	Velocity of dust particles (m${}^{2}$ s${}^{-1}$)
**Greek**
Ω	Angular velocity, (s${}^{-1}$)
$\rho_{f}$	Density of fluid, (kg m${}^{-3}$)
$\rho_{p}$	Density of dust particles, (kg m${}^{-3}$)
$\mu_{f}$	Dynamic viscosity (N s m${}^{-2}$)
$\sigma_{nf}$	Electrical conductivity, (kg${}^{-1}$ m${}^{3}$ A${}^{2}$)
$\lambda_{1}$	Heat flux relaxation time
$\nu_{nf}$	Kinematic viscosity of nanofluid, (m${}^{2}$ s${}^{-1}$)
$\beta_{m}$	Maxwell parameter
η	Similarity variable
$\tau_{T}$	Thermal equilibrium time

## References

[1] Wang J., Tan G., Wang J., Feng L.F. (2022). Numerical study on flow, heat transfer and mixing of highly viscous non-newtonian fluid in Sulzer mixer reactor. Int. J. Heat Mass Transf..

[2] Wang X., Bao L., Wen J., Dini D., Zhang J., Sun L., Yang W., Zhou F., Liu W. (2022). Anomalous boundary behavior of non-Newtonian fluids on amphiphobic surfaces. Tribol. Int..

[3] Megahed A.M., Abbas W. (2022). Non-Newtonian Cross fluid flow through a porous medium with regard to the effect of chemical reaction and thermal stratification phenomenon. Case Stud. Therm. Eng..

[4] Rashidi M.M., Chamkha A., Keimanesh M. (2011). Application of Multi-Step Differential Transform Method on Flow of a Second-Grade Fluid over a Stretching or Shrinking Sheet. Am. J. Comput. Math..

[5] Sarada K., Gowda R.J.P., Sarris I.E., Kumar R.N., Prasannakumara B.C. (2021). Effect of magnetohydrodynamics on heat transfer behaviour of a non-Newtonian fluid flow over a stretching sheet under local thermal non-equilibrium condition. Fluids.

[6] Bilal M., Urva Y. (2021). Analysis of non-Newtonian fluid flow over fine rotating thin needle for variable viscosity and activation energy. Arch. Appl. Mech..

[7] Maleki H., Safaei M.R., Alrashed A.A., Kasaeian A. (2019). Flow and heat transfer in non-Newtonian nanofluids over porous surfaces. J. Therm. Anal. Calorim..

[8] Maleki H., Safaei M.R., Togun H., Dahari M. (2019). Heat transfer and fluid flow of pseudo-plastic nanofluid over a moving permeable plate with viscous dissipation and heat absorption/generation. J. Therm. Anal. Calorim..

[9] Jamshed W., Devi S.U., Goodarzi M., Prakash M., Nisar K.S., Zakarya M., Abdel-Aty A.H. (2021). Evaluating the unsteady Casson nanofluid over a stretching sheet with solar thermal radiation: An optimal case study. Case Stud. Therm. Eng..

[10] Alazwari M.A., Abu-Hamdeh N.H., Goodarzi M. (2021). Entropy Optimization of First-Grade Viscoelastic Nanofluid Flow over a Stretching Sheet by Using Classical Keller-Box Scheme. Mathematics.

[11] Jama M., Singh T., Gamaleldin S.M., Koc M., Samara A., Isaifan R.J., Atieh M.A. (2016). Critical review on nanofluids: Preparation, characterization, and applications. J. Nanomater..

[12] Revanna Lalitha K., Veeranna Y., Thimmappa Sreenivasa G., Ashok Reddy D. (2022). Active and passive control of nanoparticles in ferromagnetic Jeffrey fluid flow. Heat Transf..

[13] Acharya N., Mabood F., Shahzad S., Badruddin I. (2022). Hydrothermal variations of radiative nanofluid flow by the influence of nanoparticles diameter and nanolayer. Int. Commun. Heat Mass Transf..

[14] Ali L., Ali B., Ghori M.B. (2022). Melting effect on Cattaneo–Christov and thermal radiation features for aligned MHD nanofluid flow comprising microorganisms to leading edge: FEM approach. Comput. Math. Appl..

[15] Ahmad L., Irfan M., Javed S., Khan M.I., Khan M.R., Niazi U.M., Alqarni A.O., El-Zahar E.R. (2022). Influential study of novel microorganism and nanoparticles during heat and mass transport in Homann flow of visco-elastic materials. Int. Commun. Heat Mass Transf..

[16] Almaneea A. (2022). Numerical study on heat and mass transport enhancement in MHD Williamson fluid via hybrid nanoparticles. Alex. Eng. J..

[17] Khan S.A., Eze C., Lau K.T., Ali B., Ahmad S., Ni S., Zhao J. (2022). Study on the novel suppression of heat transfer deterioration of supercritical water flowing in vertical tube through the suspension of alumina nanoparticles. Int. Commun. Heat Mass Transf..

[18] Ahmad S., Younis J., Ali K., Rizwan M., Ashraf M., Abd El Salam M.A. (2022). Impact of Swimming Gyrotactic Microorganisms and Viscous Dissipation on Nanoparticles Flow through a Permeable Medium: A Numerical Assessment. J. Nanomater..

[19] Nawaz M., Madkhali H.A., Haneef M., Alharbi S.O., Alaoui M. (2021). Numerical study on thermal enhancement in hyperbolic tangent fluid with dust and hybrid nanoparticles. Int. Commun. Heat Mass Transf..

[20] Zhang X.H., Abidi A., Ahmed A.E.S., Khan M.R., El-Shorbagy M., Shutaywi M., Issakhov A., Galal A.M. (2021). MHD stagnation point flow of nanofluid over a curved stretching/shrinking surface subject to the influence of Joule heating and convective condition. Case Stud. Therm. Eng..

[21] Koriko O.K., Shah N.A., Saleem S., Chung J.D., Omowaye A.J., Oreyeni T. (2021). Exploration of bioconvection flow of MHD thixotropic nanofluid past a vertical surface coexisting with both nanoparticles and gyrotactic microorganisms. Sci. Rep..

[22] Dadheech P.K., Agrawal P., Sharma A., Dadheech A., Al-Mdallal Q., Purohit S.D. (2021). Entropy analysis for radiative inclined MHD slip flow with heat source in porous medium for two different fluids. Case Stud. Therm. Eng..

[23] Abo-Dahab S., Abdelhafez M., Mebarek-Oudina F., Bilal S. (2021). MHD Casson nanofluid flow over nonlinearly heated porous medium in presence of extending surface effect with suction/injection. Indian J. Phys..

[24] Nazeer M., Hussain F., Khan M.I., Rehman A., El-Zahar E.R., Chu Y.M., Malik M. (2022). Theoretical study of MHD electro-osmotically flow of third-grade fluid in micro channel. Appl. Math. Comput..

[25] Salahuddin T., Khan M.H.U., Khan M., Al Alwan B., Amari A. (2022). An analysis on the flow behavior of MHD nanofluid with heat generation. Fuel.

[26] Rehman K.U., Shatanawi W., Abodayeh K. (2022). A group theoretic analysis on heat transfer in MHD thermally slip Carreau fluid subject to multiple flow regimes (MFRs). Case Stud. Therm. Eng..

[27] Zhang J., Anjal H.A., Msmali A., Wang F., Nofal T.A., Selim M.M. (2022). Heat transfer of nanomaterial with involve of MHD through an enclosure. Case Stud. Therm. Eng..

[28] Ali L., Ali B., Liu X., Iqbal T., Zulqarnain R.M., Javid M. (2022). A comparative study of unsteady MHD Falkner-Skan wedge flow for non-Newtonian nanofluids considering thermal radiation and activation energy. Chin. J. Phys..

[29] Ali L., Ali B., Liu X., Ahmed S., Shah M.A. Analysis of bio-convective MHD Blasius and Sakiadis flow with Cattaneo-Christov heat flux model and chemical reaction. Chin. J. Phys..

[30] Shah N.A., Wakif A., Shah R., Yook S.J., Salah B., Mahsud Y., Hussain K. (2021). Effects of fractional derivative and heat source/sink on MHD free convection flow of nanofluids in a vertical cylinder: A generalized Fourier’s law model. Case Stud. Therm. Eng..

[31] Khan M., Hafeez A., Ahmed J. (2021). Impacts of non-linear radiation and activation energy on the axisymmetric rotating flow of Oldroyd-B fluid. Phys. A: Stat. Mech. Its Appl..

[32] Krishna M.V., Ahamad N.A., Aljohani A. (2021). Thermal radiation, chemical reaction, Hall and ion slip effects on MHD oscillatory rotating flow of micro-polar liquid. Alex. Eng. J..

[33] Zubair T., Usman M., Nisar K.S., Hamid M., Mahmoud E.E., Yahia I. (2022). Investigation of shape effects of Cu-nanoparticle on heat transfer of MHD rotating flow over nonlinear stretching sheet. Alex. Eng. J..

[34] Hayat T., Ahmad M.W., Khan S.A., Alsaedi A. (2021). Rotating flow of viscous nanomaterial with radiation and entropy generation. Adv. Mech. Eng..

[35] Khan M.N., Nadeem S. (2021). A comparative study between linear and exponential stretching sheet with double stratification of a rotating Maxwell nanofluid flow. Surfaces Interfaces.

[36] Yacob N.A., Dzulkifli N.F., Salleh S.N.A., Ishak A., Pop I. (2022). Rotating flow in a nanofluid with CNT nanoparticles over a stretching/shrinking surface. Mathematics.

[37] Ali B., Thumma T., Habib D., Salamat N., Riaz S. (2022). Finite element analysis on transient MHD 3D rotating flow of Maxwell and tangent hyperbolic nanofluid past a bidirectional stretching sheet with Cattaneo Christov heat flux model. Therm. Sci. Eng. Prog..

[38] Ali L., Liu X., Ali B., Din A., Al Mdallal Q. (2021). The function of nanoparticle’s diameter and Darcy-Forchheimer flow over a cylinder with effect of magnetic field and thermal radiation. Case Stud. Therm. Eng..

[39] Kotresh M.J., Ramesh G.K., Shashikala V.K.R., Prasannakumara B.C. (2021). Assessment of Arrhenius activation energy in stretched flow of nanofluid over a rotating disc. Heat Transf..

[40] Mahanthesh B., Makinde O.D., Gireesha B.J., Krupalakshmi K.L., Animasaun I.L. (2018). Two-phase flow of dusty Casson fluid with Cattaneo-Christov heat flux and heat source past a cone, wedge and plate. Defect and Diffusion Forum.

[41] Mahanthesh B., Mackolil J., Radhika M., Al-Kouz W., Siddabasappa (2021). Significance of quadratic thermal radiation and quadratic convection on boundary layer two-phase flow of a dusty nanoliquid past a vertical plate. Int. Commun. Heat Mass Transf..

[42] Mahanthesh B., Animasaun I., Rahimi-Gorji M., Alarifi I.M. (2019). Quadratic convective transport of dusty Casson and dusty Carreau fluids past a stretched surface with nonlinear thermal radiation, convective condition and non-uniform heat source/sink. Phys. A Stat. Mech. Its Appl..

[43] Mahanthesh B., Shashikumar N.S., Gireesha B.J., Animasaun I.L. (2019). Effectiveness of Hall current and exponential heat source on unsteady heat transport of dusty TiO2-EO nanoliquid with nonlinear radiative heat. J. Comput. Des. Eng..

[44] Gireesha B., Mahanthesh B., Thammanna G., Sampathkumar P. (2018). Hall effects on dusty nanofluid two-phase transient flow past a stretching sheet using KVL model. J. Mol. Liq..

[45] Mahanthesh B., Gireesha B. (2018). Thermal Marangoni convection in two-phase flow of dustyCasson fluid. Results Phys..

[46] Gireesha B.J., Mahanthesh B., Makinde O.D., Muhammad T. (2018). Effects of Hall current on transient flow of dusty fluid with nonlinear radiation past a convectively heated stretching plate. Defect and Diffusion Forum.

[47] Rashid S., Hayat T., Qayyum S., Ayub M., Alsaedi A. (2019). Three-dimensional rotating Darcy–Forchheimer flow with activation energy. Int. J. Numer. Methods Heat Fluid Flow.

[48] Tayyab M., Siddique I., Jarad F., Ashraf M.K., Ali B. (2022). Numerical solution of 3D rotating nanofluid flow subject to Darcy-Forchheimer law, bio-convection and activation energy. S. Afr. J. Chem. Eng..

[49] Shah N.A., Wang S., Elnaqeeb T., Qi H. (2021). Soret and memory effects on unsteady MHD natural convection heat and mass transfer flow in a porous medium with Newtonian heating. J. Porous Media.

[50] Ali B., Siddique I., Ahmadian A., Senu N., Ali L., Haider A. (2022). Significance of Lorentz and Coriolis forces on dynamics of water based silver tiny particles via finite element simulation. Ain Shams Eng. J..

[51] Shah N.A., Animasaun I., Chung J.D., Wakif A., Alao F., Raju C. (2021). Significance of nanoparticle’s radius, heat flux due to concentration gradient, and mass flux due to temperature gradient: The case of Water conveying copper nanoparticles. Sci. Rep..

[52] Rehman S.U., Mariam A., Ullah A., Asjad M.I., Bajuri M.Y., Pansera B.A., Ahmadian A. (2021). Numerical computation of buoyancy and radiation effects on MHD micropolar nanofluid flow over a stretching/shrinking sheet with heat source. Case Stud. Therm. Eng..

[53] Wang F., Asjad M.I., Ur Rehman S., Ali B., Hussain S., Gia T.N., Muhammad T. (2021). MHD Williamson Nanofluid Flow over a Slender Elastic Sheet of Irregular Thickness in the Presence of Bioconvection. Nanomaterials.

[54] Namburu P., Kulkarni D., Dandekar A., Das D. (2007). Experimental investigation of viscosity and specific heat of silicon dioxide nanofluids. Micro Nano Lett..

